# Evaluation of the Quality and Suitability of Self‐Collected Vaginal and Urine Samples for Human Papillomavirus Testing: A Prospective Matched Study

**DOI:** 10.1111/1471-0528.70170

**Published:** 2026-02-04

**Authors:** Kim Chu, Sofia Vidali, Anna Parberry, Michelle Saull, Krishna Patel, Hannah Mohy‐Eldin, Laura White, Adam Brentnall, Peter Sasieni, Rhian Gabe, Ranjit Manchanda, Jack Cuzick, Belinda Nedjai

**Affiliations:** ^1^ Barts CTU, Wolfson Institute of Population Health Queen Mary University of London London UK; ^2^ Wolfson Institute of Population Health Queen Mary University of London London UK; ^3^ Colposcopy Department Royal London Hospital London UK; ^4^ Department of Gynaecological Oncology Barts Health NHS Trust London UK

**Keywords:** cervical cancer, HPV, self‐sampling, stability, urine, vaginal swab

## Abstract

**Objective:**

To evaluate the analytical suitability of different storage and laboratory processes of self‐samples for an HPV assay.

**Design:**

Prospective matched study.

**Setting:**

Royal London Hospital Colposcopy Clinic.

**Population:**

One hundred seventy seven patients aged 25–65 years referred to colposcopy due to their screening results (abnormal cytology or recurrent HPV infection).

**Methods:**

Each participant provided a first void urine sample (10 mL/20 mL, Collipee), two vaginal self‐samples (Copan FLOQSwabs transported ‘dry’ and ‘wet’), and a clinician‐collected cervical sample. Samples were processed immediately or after 1 or 2 weeks stored at room temperature. HPV testing used BD Onclarity.

**Main Outcome Measures:**

Genomic DNA Quality Score (GQS), detection of HPV, and HPV cycle threshold (Ct) values.

**Results:**

DNA quality of dry samples was not significantly lower than wet samples when resuspended within 2 weeks (median GQS dry vs. wet: 3.35 vs. 3.41, immediately; 3.00 vs. 3.14, 1 week; 3.45 vs. 2.78, 2 weeks; all *p* [one‐sided] > 0.05). Urine samples had lower HPV positivity compared to other sample types and had higher HPV Ct values (median 30 vs. 27 for dry/wet/clinician samples).

**Conclusion:**

Dry self‐samples from a Copan FLOQSwab taken in clinic are likely to have sufficient DNA quality and accuracy for HPV testing compared with wet self‐samples and clinician‐collected samples if resuspension takes place up to 2 weeks, with storage at room temperature, and using the BD Onclarity assay. Urine samples using the Colli‐pee device are likely to be less sensitive for HPV detected from clinician‐collected samples.

## 1 Introduction

Cervical screening programmes have reduced the population burden of cervical cancer [1, 2, 3, 4, 5, 6]. The most effective screening test detects human papillomavirus (HPV), which causes over 99% of cervical cancers [6, 7]. In many places, a cervical sample is taken for testing by a healthcare practitioner, but some settings offer vaginal self‐sampling. Self‐sampling may address barriers to traditional clinician screening and improve user experience [8, 9, 10]. However, there is uncertainty about the clinical effectiveness of self‐sampling compared with clinician sampling for detecting high‐grade cervical intraepithelial neoplasia (CIN grade 2/3), the main target of screening. While early analyses suggested self‐ and clinician‐collected sampling was equivalent, this has not always been consistent with real‐world implementation data [11]. For example, when self‐sampling was introduced in the Netherlands, observational analysis suggested that, in a worst case scenario, primary use of self‐sampling could be associated with up to a potential 25% loss in sensitivity [12]. The authors and others identified storage medium as an issue with implementation [12]. In particular, using 20 mL for a self‐collection device (the standard for a clinician‐collected device) led to a loss of signal for the HPV assay. This highlights the importance of studies to validate analytical suitability of the complete process or workflow by which self‐sampling is intended to be offered before offering the test at scale. Clinician‐collected samples are taken under relatively standard conditions with consistent methods for sample storage and transportation. In contrast, self‐collected samples taken at home vary widely in collection method, the conditions they're exposed to, and transportation time. Verifying the impact of such issues is therefore important.

Laboratory synthetic studies have evaluated reliability and stability of HPV detection under varying conditions, including transport medium, resuspension volume, time to testing and storage temperature [13, 14, 15, 16]. Experiments using proxies for vaginal self‐samples found that length of stability depends on the device and storage conditions [15, 16]. There are relatively few paired comparative studies on real‐world stability and reliability of different HPV self‐sample workflows in the literature. Some studies have evaluated HPV agreement between vaginal samples transported wet vs. dry. Generally, wet samples show slightly greater stability than dry ones, which may be associated with lower HPV detection rates in dry samples [17, 18, 19, 20, 21]. In some settings, dry samples, while logistically advantageous, can produce comparable results [17, 18, 19, 20, 21, 22]. Studies evaluating urine samples demonstrated the importance of using specialised devices to capture first void urine, rather than standard urine pots [23, 24, 25, 26]. Nevertheless, even the specialised device, Colli‐Pee, using first void urine samples missed some HPV infections when tested using standard cycle threshold (Ct) cutoffs on HPV assays [27].

### 1.1 Statement of Study Objective

This study evaluates analytical suitability of self‐collection methods for HPV testing, specifically comparing wet and dry vaginal samples, after storage at room temperature (RT) for up to 2 weeks in the laboratory, using the BD Onclarity assay. Therefore, a secondary objective was to evaluate the distribution of Ct values by device and between workflows.

## 2 Methods

### 2.1 Study Design

In this prospective matched study, consenting participants were asked to provide one self‐collected urine sample, followed by two self‐collected vaginal samples (one dry, one wet in a transport medium; order not fixed), and finally a clinician‐collected cervical sample at the Royal London Hospital (RLH) Colposcopy clinic. In addition, participants were asked to provide an optional home‐collected urine sample (sent back to the laboratory within a week). Optional at‐home collection was included to assess the feasibility and return rate of home urine sampling for planning future studies. Participants received standardised written instructions and diagrammatic illustrations detailing the self‐collection procedure, with no in‐person demonstration (Figure S1). Adherence was defined as opening the sealed vaginal swab packages and placing one swab into the wet storage medium. Participants were randomised 1:1:1:1 based on recruitment order, following a block‐randomised list (block size 50) to receive one of the following packs: 2 FloqSwabs, BD medium, 10 mL Colli‐Pee device; 2 FloqSwabs, BD medium, 20 mL Colli‐Pee device; 2 FloqSwabs, Copan MSwab medium, 10 mL Colli‐Pee device; 2 FloqSwabs, Copan MSwab medium, 10 mL Colli‐Pee device. The optional urine sample was a 3 mL Colli‐Pee device. Once samples from the clinic arrived in the lab, samples were allocated resuspension/processing time groups (immediately, after 1 week at RT, after 2 weeks at RT) on an ad‐hoc basis based on lab operational capacity. Dry samples were resuspended and tested at their assigned time group, with all other sample types.

### 2.2 Setting and Participants

Patients aged 25–65 years referred to colposcopy at the RLH Colposcopy Clinic due to their screening results (abnormal cytology or recurrent HPV infection) were recruited between 29th April and 21st October 2024. Abnormal cytology was defined according to the UK National Health Service Cervical Screening Programme guidelines as any result reported as borderline nuclear changes (squamous or endocervical) or worse, including low‐grade dyskaryosis, high‐grade dyskaryosis, or results suspicious for malignancy. Exclusion criteria were pregnancy, history of excisional or ablative treatment for CIN within the last 3 years (not including application of haemostatic agents post punch biopsy), and people without a cervix.

### 2.3 Variables

The primary outcome was Genomic DNA Quality Score (GQS), measured using the LabChip GX (PerkinElmer). GQS ranges from 0 to 5, with 5 being the highest quality and GQS ≥ 1.5 being acceptable sample quality. The GQS is derived from the size distribution of the DNA; increasing degradation is reflected in smaller‐sized fragments of genomic DNA. The adequacy threshold (≥ 1.5) was defined based on findings from a prior study (Predictors 5.1, P5.1) conducted in the same setting, in which 14% of self‐collected dry samples and 1% of self‐collected wet samples had GQS ≤ 1.5 [19]. This study aimed to develop a self‐sampling and laboratory workflow that achieves DNA quality exceeding that of the previously evaluated dry device; therefore, GQS ≥ 1.5 was used as a benchmark indicator of adequate sample quality. All samples were tested for high‐risk HPV (hrHPV) using the BD Onclarity HPV Assay, which measures β‐globin (assay's internal control) and detects 14 hrHPV genotypes: six individually (16, 18, 31, 45, 51 and 52) and three grouped channels ([33, 58]; [56, 59, 66]; [35, 39, 68]). Urine samples were processed according to the BD Viper manufacturer's instructions. Samples were inverted several times to ensure homogenisation, and a starting volume of 2.5–3.0 mL was transferred into BD specimen tubes approved for use on the BD Viper system. No centrifugation step was performed prior to genotyping. Wet vaginal samples were homogenised by repeated pipetting, and 1 mL of sample was transferred into BD Onclarity HPV LBC Dilution Tube in accordance with the manufacturer's protocol. Dry vaginal samples were resuspended in the selected transport medium (BD or Copan), then homogenised, and 1 mL of the resulting suspension was added to the BD Onclarity HPV LBC Dilution Tube following manufacturer's instructions. Cervical samples were homogenised by pipetting, and 1 mL of sample was transferred into the BD Onclarity HPV LBC Dilution Tube as per the BD Viper manufacturer's guidelines. The manufacturer's HPV positivity threshold was a Ct value ≤ 38.3 for HPV16 or ≤ 34.2 for all other genotypes. A secondary outcome was the Ct score defined as the minimum HPV Ct value across the HPV types/channels. When an HPV Ct value was not detected, an ad hoc value of 40 was assigned (slightly higher than manufacturer's Ct threshold) for use in Ct analysis as a continuous variable. All such samples were HPV negative (*n* = 82). DNA quantity was measured using the Qubit Fluorometer (ThermoFisher Scientific). Time to resuspension in days was calculated as resuspension date minus sample collection date. For each sample, GQS, HPV positivity, Ct score and DNA quantity were measured at the assigned sample time point. Participants' postcodes were used to obtain the 2019 index of multiple deprivation (IMD) from the Ministry of Housing, Communities & Local Government website [28, 29]. IMD was grouped into quintiles (1st = most deprived). Age groups were < 30, 30–44 and ≥ 45 years. DNA quantity was transformed to log (1 + DNA quantity) to address skewness in plots. CIN2+ was defined as histologically confirmed CIN grade 2 or higher.

### 2.4 Study Size

An earlier study (Predictors 5.1, P5.1) in a referral setting found a mean difference in DNA quality score between paired Wet Dacron and Dry FloqSwab samples of 0.524, standard deviation = 0.907, with an approximate normal distribution [19]. The main objective of our study was to compare the DNA quality between wet and dry self‐samples, controlling for time to resuspension of dry samples. To detect the same size difference that was observed in P5.1, 102 people (34 per time group) were needed to obtain 90% power when testing superiority in each resuspension time group using a *t*‐test at a (unadjusted) 5% level. The study was powered to compare DNA quality between dry and wet vaginal self‐samples and was not designed to formally power comparisons between processing‐time groups.

### 2.5 Statistical Methods

For the primary analysis, median GQS differences between paired wet and dry vaginal samples were assessed using the Wilcoxon Signed Rank test. Comparisons across processing time groups were considered exploratory and descriptive; therefore, no formal adjustment for multiple comparisons was applied, and nominal *p*‐values should be interpreted with caution. Summary statistics were evaluated for DNA quality, Ct score, HPV positivity, and DNA quantity for each sample workflow, with boxplots for continuous variables. Dry samples were analysed by resuspension time group and overall. HPV concordance between paired samples was calculated. For HPV discordant pairs, summary statistics of the distribution of Ct scores of the HPV positive samples were calculated. Scatterplots and Pearson correlation coefficients of Ct values, GQS, and DNA quantity between paired wet and dry samples and paired clinician and dry samples were produced. For each sample type, sensitivity, specificity, PPV, and negative predictive value (NPV) for detection of CIN2+ were calculated. These analyses were exploratory, as the study was not powered for lesion‐based outcomes.

## 3 Results

Between 29th April and 21st October 2024, on days when the number of people approached was recorded, 444 were approached, with 155 (35%) consenting (Figure S2). On these recruitment days, the primary reasons for non‐consent were unknown (34%; 99/289), ineligibility (32%; 92/289), and unwillingness (26%; 74/289). On days where the number approached was not recorded, 22 people consented. Overall, 177 people consented. After excluding two ineligible consenting people, 175 people were included in the study, providing 793 sufficient samples for analysis. Of the 175 participants, 174 (99.4%) adhered by giving dry and wet vaginal samples, 167 (95.4%) gave dry, wet, and urine samples, 173 (98.9%) gave dry, wet, and clinician samples, and 166 (94.9%) had all four samples.

The median age was 30 years (interquartile range (IQR): 26–35; range 25–62) (Table S1). Median time to colposcopy from screening was 2.1 months (IQR: 1.7–2.4, range 0.7–9.0), and 75% were referred following HPV positive and abnormal cytology at the baseline screen (Screen 1, Table S1). One hundred twenty five participants (71.4%) were from the two most deprived IMD quintiles.

For the primary analysis, of the 174 paired vaginal samples, 59 were processed immediately (TG1), 72 after 1 week (TG2), and 43 after 2 weeks (TG3) (Table 1). Across all time groups, dry and wet samples had a median GQS ≥ 3.00 and GQS ≥ 2.74, respectively, with no (one‐sided) significant differences between them. The increase in GQS observed for dry samples at week 2 likely represents random variation and should not be interpreted as evidence that dry samples outperform wet samples.

**TABLE 1:** DNA quality score by vaginal sample type and resuspension timing group, and the difference in DNA quality score between dry and wet vaginal samples (dry‐wet) tested using Wilcoxon Signed Rank test.

Resuspension group	Number of women	Median (IQR) DNA quality score (Dry)	Median (IQR) DNA quality score (Wet)	Median difference (Dry—Wet)	95% CI for median difference (Dry–Wet)	*p*
Time group 1 (immediate)	59	3.35 (3.00, 3.67)	3.41 (2.99, 3.81)	−0.06	−0.24, 0.14	0.49
Time group 2 (week 1)	72	3.00 (2.79, 3.40)	3.14 (2.81, 3.55)	−0.05	−0.32, 0.21	0.67
Time group 3 (week 2)	43	3.45 (2.75, 3.84)	2.78 (2.26, 3.24)	0.64	0.27, 0.98	0.001

Abbreviations: CI, confidence interval; IQR, interquartile range.

All sample types had a median GQS ≥ 2.85, with most exceeding the adequacy threshold of 1.5 (Table 2, Figure S3). Dry samples showed the highest adequacy (97.7%), then wet (96.6%), clinician (95.4%), and urine (92.3%). GQS correlation between dry and wet samples was low in TG1 and absent in TG2 and TG3. Correlation between clinician and dry samples is in the Supporting Information (Figures S4 and S5).

**TABLE 2:** Summary statistics of DNA quality, Ct score, S5 score, HPV positivity, DNA quantity by sample type, and resuspension time group.

	Sample types (subgroups combined)				Dry sample resuspension group		
	Wet	Urine	Clinician	Dry	Time group 1 (immediate)	Time group 2 (week 1)	Time group 3 (week 2)
*N*	174	168	174	175	60	72	43
Median (IQR) [range]							
DNA quality	3.13 (2.69, 3.60) [0.42, 4.35]	2.85 (2.40, 3.28) [0.02, 4.15]	2.94 (2.57, 3.30) [0.38, 4.23]	3.26 (2.88, 3.67) [0.00, 4.40]	3.38 (3.00, 3.67) [0.00, 4.34]	3.00 (2.79, 3.40) [0.54, 4.40]	3.45 (2.75, 3.84) [1.21, 4.39]
Unknown *N* (%)	0 (0.0)	0 (0.0)	0 (0.0)	0 (0.0)	0 (0.0)	0 (0.0)	0 (0.0)
Lowest HPV Ct score	27.0 (23.5, 35.5) [17.8, 40.0]	30.3 (26.7, 35.6) [20.0, 40.0]	27.2 (23.7, 32.1) [17.0, 40.0]	27.3 (24.5, 32.1) [13.7, 40.0]	27.2 (24.6, 31.4) [17.6, 40.0]	27.5 (24.6, 32.2) [18.2, 40.0]	27.0 (24.5, 33.0) [13.7, 39.1]
Unknown *N* (%)	0 (0.0)	2 (1.2)	0 (0.0)	0 (0.0)	0 (0.0)	0 (0.0)	0 (0.0)
DNA quantity	21.0 (10.7, 36.4) [0.8, 226.0]	21.8 (9.3, 41.5) [0.1, 296.0]	15.3 (6.9, 39.3) [0.1, 320.0]	17.0 (6.6, 33.4) [0.5, 142.0]	16.4 (6.3, 25.4) [0.5, 79.0]	14.6 (6.3, 24.6) [0.5, 115.0]	24.6 (10.5, 41.0) [2.0, 142.0]
Unknown *N* (%)	2 (1.1)	5 (3.0)	4 (2.3)	2 (1.1)	0 (0.0)	2 (2.8)	0 (0.0)
*n*/*N* (%)							
HPV positive	140/174 (80.5)	114/166 (68.7)	139/174 (79.9)	147/175 (84.0)	51/60 (85.0)	60/72 (83.3)	36/43 (83.7)
Unknown	0 (0.0)	2 (1.2)	0 (0.0)	0 (0.0)	0 (0.0)	0 (0.0)	0 (0.0)
Adequate DNA quality (GQS ≥ 1.5)	168/174 (96.6)	155/168 (92.3)	166/174 (95.4)	171/175 (97.7)	59/60 (98.3)	70/72 (97.2)	42/43 (97.7)
Unknown	0 (0.0)	0 (0.0)	0 (0.0)	0 (0.0)	0 (0.0)	0 (0.0)	0 (0.0)

Abbreviations: GQS, genomic DNA quality score; IQR, interquartile range.

HPV testing was unsuccessful for two urine samples (failed internal control). HPV positivity was comparable between clinician‐collected (79.9% (139/174), 95% CI: 73.3% to 85.2%) and vaginal samples (dry = 84.0% (147/175), 95% CI: 77.8% to 88.7%; wet = 80.5% (140/174), 95% CI: 73.9% to 85.7%). But only 68.7% (115/166, 95% CI: 61.9% to 75.8%) of urine samples were HPV positive.

Table 2 shows urine samples exhibited the highest median HPV Ct value of 30.3 (IQR: 26.7, 35.6) in contrast to lower median Ct values in wet (27.0; IQR: 23.5, 35.5), clinician‐collected (27.2; IQR: 23.7, 32.1), and dry samples (27.3; IQR: 24.5, 32.1). Among dry samples, HPV positivity rates and average Ct values remained consistent across all time points.

**FIGURE 1:**
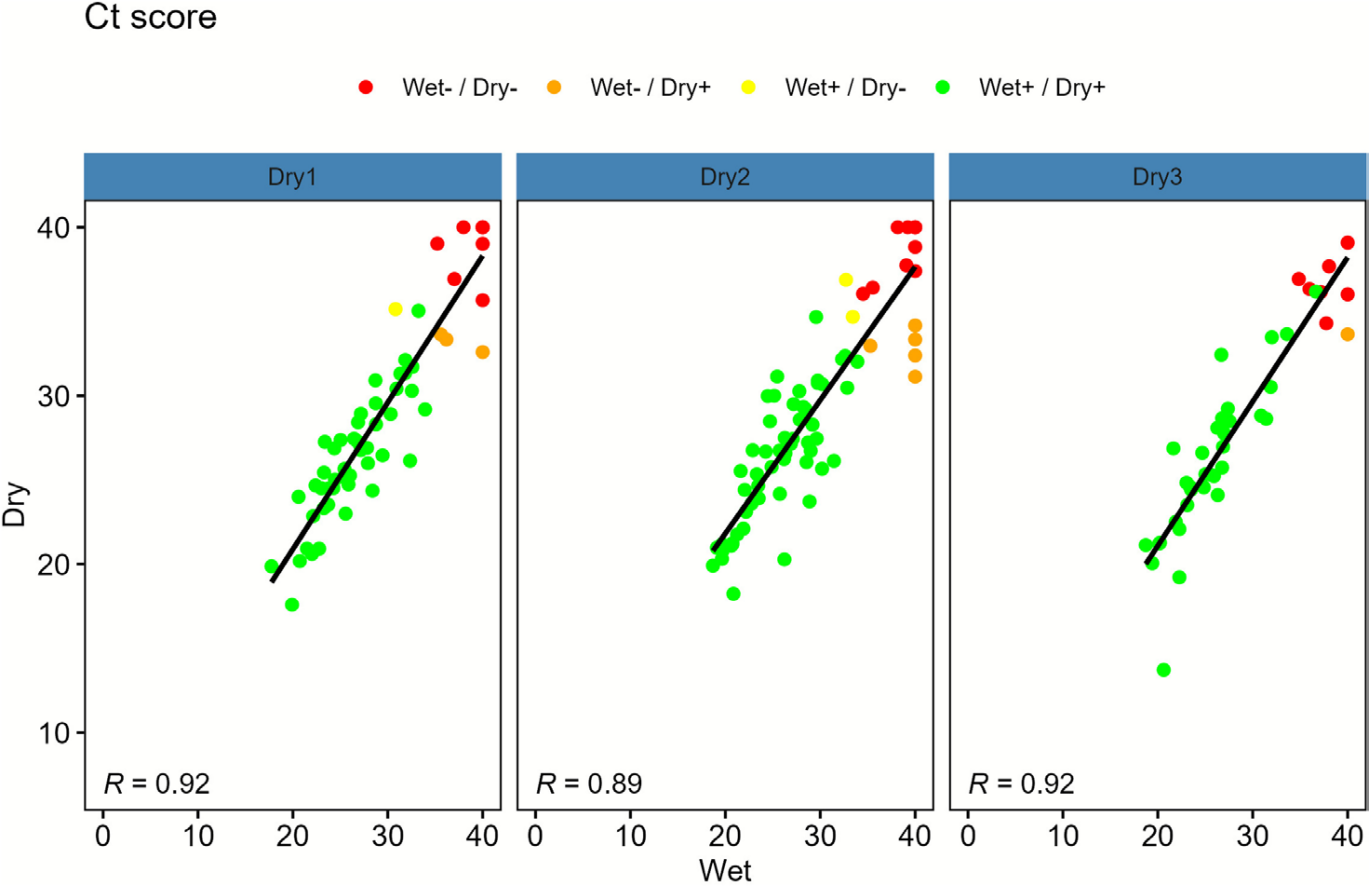
Scatterplots of Ct score with a regression line and Pearson correlation coefficients for wet versus paired dry sample (by resuspension time group).

**FIGURE 2:**
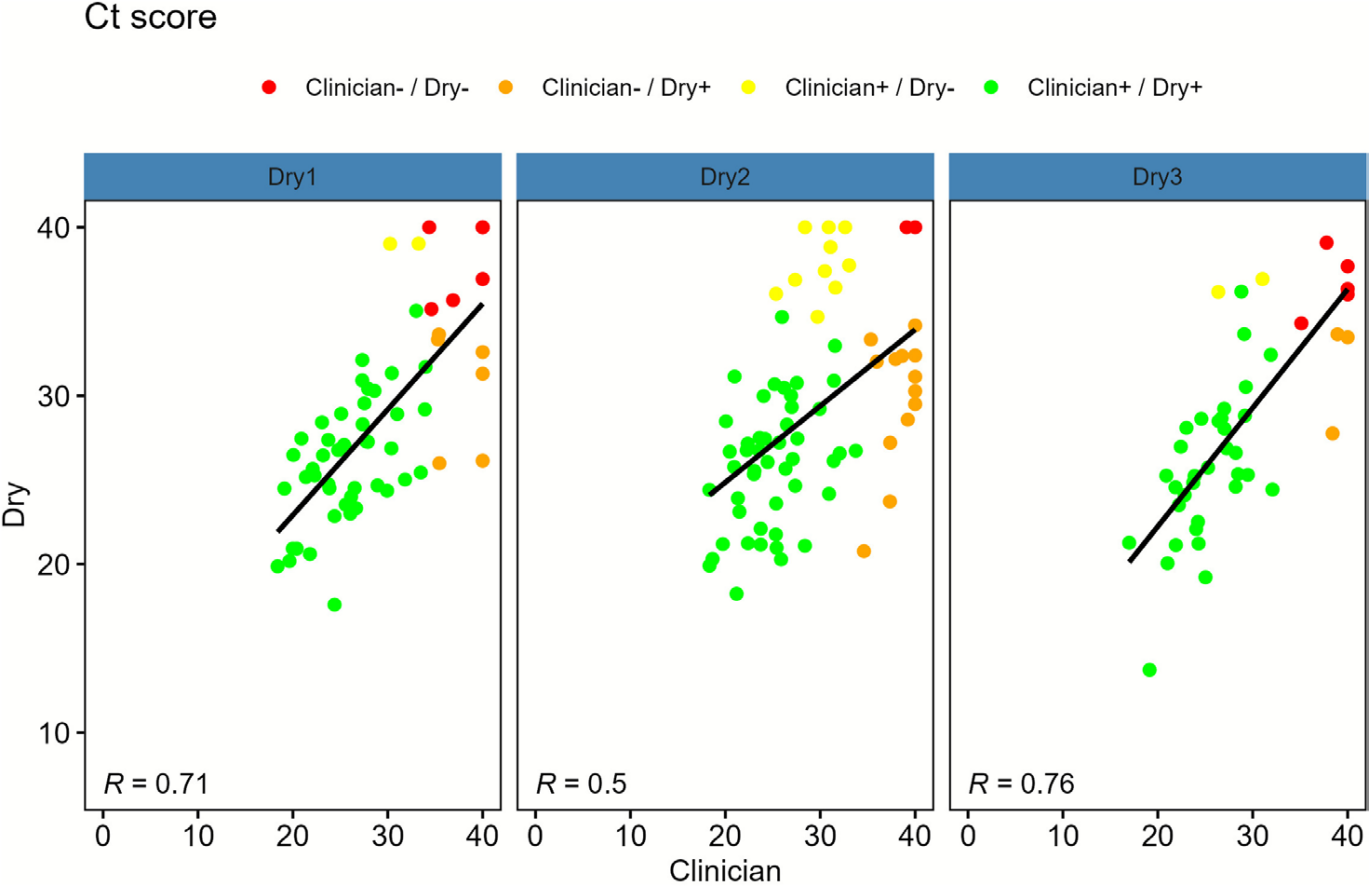
Scatterplots of Ct score with a regression line and Pearson correlation coefficients for clinician versus paired dry sample (by resuspension time group).

Agreement between dry and wet samples was high across all time groups (Table S3). Concordant HPV positive pairs had lower median Ct values than discordant pairs (Table S4). To evaluate correlation in viral load between samples, the Ct score was used. Correlation between dry and wet samples was strong in all time groups (TG1: *R* = 0.92; TG2: *R* = 0.89; TG3: *R* = 0.92) (Figure 1), and moderate between dry and clinician samples (TG1: *R* = 0.71; TG2: *R* = 0.50; TG3: *R* = 0.76) (Figure 2).

Urine samples yielded the highest average DNA quantity (21.8; IQR: 9.3, 41.5), while clinician samples yielded the lowest (15.3; IQR: 6.9, 39.3) (Table 2). Among vaginal samples, dry samples had lower DNA (17.0; IQR: 6.6, 33.4) compared with wet samples (21.0; IQR: 10.7, 36.4), with DNA quantity in dry samples declining over time (TG1: 17.0; TG2: 16.4; TG3: 14.6). DNA quantity correlations for wet vs. dry and clinician vs. dry are reported in the Supporting Information (Figures S6 and S7).

There were 25 CIN2+ cases. For dry samples, CIN2+ detection sensitivity decreased slightly as time to resuspension increased (TG1: 100.0% (95% CI: 59.0–100.0); TG2: 91.7% (95% CI: 61.5–99.8); TG3: 83.3% (95% CI: 35.9–99.6)). Clinician and both vaginal sample types had the highest sensitivity (92.0%, 95% CI: 74.0–99.0), while urine was 79.2% (95% CI: 57.8–92.9). Specificity, PPV and NPV are in Table S5.

## 4 Discussion

### 4.1 Main Findings

This study evaluated the quality and diagnostic performance of self‐collected vaginal samples (dry and wet), urine samples and clinician‐collected cervical samples for HPV testing, focusing on vaginal samples and the effect of delayed processing of dry samples stored at RT. Both dry and wet vaginal samples collected using the Copan FLOQSwab provided sufficient DNA quality and reliable HPV detection using the BD assay compared with clinician samples, even when stored at RT and processed up to 2 weeks after laboratory arrival. All sample types achieved high rates of DNA adequacy, with 95.5% of samples meeting the predefined threshold.

Among those previously testing HPV positive on a clinician‐collected sample, urine samples collected using the Colli‐Pee device had lower HPV positivity than other sample types, which had similar positivity rates to each other in this population. Additionally, in this population, urine samples exhibited the highest median Ct values, suggesting lower viral load or DNA concentration, highlighting potential need for urine‐specific thresholds.

Dry vaginal samples maintained stable HPV positivity and Ct values across all time points, indicating processing delays up to 2 weeks at RT did not affect test performance. Correlation of Ct values between wet and dry vaginal samples was high across all time groups, and agreement in HPV detection was strong. Furthermore, discordant HPV results tended to occur in cases with higher Ct values, suggesting that low viral load may underlie inconsistencies. Clinician‐collected cervical and self‐collected dry vaginal samples had similar HPV positivity but only moderate correlation across all time points, likely reflecting inherent anatomical differences between cervical and vaginal samples.

### 4.2 Strengths and Limitations

Key strengths of this study include its prospective matched design, inclusion of multiple self‐collected sample types (wet and dry vaginal and urine) alongside clinician‐collected cervical specimens, and the use of a clinically validated HPV assay [30].

A study limitation was incomplete recording of women approached, preventing full participation rate calculation and potentially introducing selection bias. Other limitations include the referral population, where HPV prevalence is higher by definition and viral loads in those testing HPV+ are likely to be higher than would be expected in a general screening population [31]. Thus, the findings are not directly generalisable to population‐based primary screening settings, where a greater proportion of infections are expected to exhibit low viral loads. Ct value distributions observed in this study are therefore unlikely to match those in true screening cohorts. Consequently, while dry self‐collected samples demonstrated analytical stability for up to 2 weeks in this population, these results might not fully translate to real‐world screening conditions, where detection of low viral load infections may be affected by other factors such as temperature variation. In addition, a risk of bias is that participants were not randomised to processing time groups, as allocation was determined by laboratory workflow. Although baseline demographic characteristics were examined and found to be broadly similar across groups, non‐random allocation may have introduced selection bias or residual confounding related to sampling day or viral load distribution. Consequently, observed differences between processing‐delay groups might not be attributable solely to processing time. Sequential collection of multiple samples may influence viral load or cell yield captured by individual devices. However, previous studies suggest sampling order does not materially impact HPV test outcomes in vaginal samples [19]. Order of vaginal self‐sampling was not recorded in this study, so we were unable to formally assess order effects; therefore, although we believe unlikely, we cannot fully exclude order effects in the current study population. Lastly, we only performed exploratory analyses and did not formally evaluate urine volume and sample media, which showed no significant differences in HPV positivity or Ct values (Tables S6 and S7). Participants were not instructed to perform genital washing prior to urine collection, and the timing of last urination was not recorded. These factors could have influenced viral load in urine and may contribute to HPV detection variability. Further, while this study suggests Copan FLOQSwabs can be used for either dry or wet transport without compromising DNA quality for up to 2 weeks when stored at RT, we note that our results apply specifically to laboratory workflows that involve RT storage for this duration. Care should be taken for generalisation to workflows involving longer storage period or exposure to extreme cold or hot temperatures. Similarly, the results may not be directly applicable to other self‐sample collection devices or HPV testing platforms, which may differ in sensitivity or sample handling requirements. A further limitation is that no published studies have examined the impact of GQS change on HPV detection. Although CIN2+ detection was assessed, the small number of cases limits statistical power and precision, so findings are exploratory rather than definitive.

### 4.3 Interpretation

Dry vaginal self‐samples had high adequacy rates for DNA quality. These findings support prior studies showing dry self‐sampling can be a robust method for nucleic acid preservation and HPV testing [11, 32, 33]. Additionally, Ct values from HPV assays may likely have relevance for cervical screening, potentially serving a role in triage strategies [34]. Also, Ct values may play a role in evaluating HPV assay performance for detecting CIN2+ in routine screening [35], and as an indirect measure to account for spectrum effects in validation studies of relative sensitivity of self‐sampling for CIN2+.

Urine samples had lower HPV positivity and higher HPV Ct values compared to the other sample types. This aligns with some previous findings indicating urine sampling (first‐void and random void) may be less sensitive to HPV detection in women positive at primary screening from a clinician‐collected HPV test, due to lower exfoliated cell content or suboptimal viral DNA concentration [36, 37, 38, 39]. Also, stratified analyses by urine volume (10 mL vs. 20 mL) did not show statistically significant differences, suggesting that volume alone is unlikely to fully explain these findings. Urine sampling is completely different than a swab; assay positivity thresholds or procedures for urine samples may need adjustment, if higher agreement with clinician or self‐collected swabs is wanted. Interestingly, in primary screening settings, some studies found HPV testing of Colli‐Pee samples had lower specificity than clinician‐collected samples [40]. Although HPV positivity rates were similar between clinician‐collected cervical samples and dry vaginal self‐samples, correlations in Ct values were only moderate (*R* = 0.50–0.76). This ‘discrepancy’ is due to the difference between a binary outcome based on a continuous measure, and correlation of the continuous measure (viral load). Previous studies have also reported that vaginal self‐samples exhibited higher Ct values than cervical samples despite comparable HPV positivity, suggesting that these differences likely reflect anatomical differences in cervical and vaginal sampling [35, 41].

## 5 Conclusion

In this referral population, dry vaginal self‐sampling using Copan FLOQSwab appears reliable for HPV testing with RT storage up to 2 weeks after sample collection. However, these findings should be interpreted with caution and cannot be directly extrapolated to population‐based primary screening programmes. Moderate Ct value differences between clinician‐collected and self‐collected samples are expected due to anatomical and sampling differences, but these do not appear to affect overall HPV detection. Urine self‐sampling, while promising for its acceptability, may require further optimisation to improve test performance or other adjustments to screening pathways that acknowledge the difference in performance. Future research in real‐world implementation within screening populations (such as at‐home tests) and using randomised processing‐delay designs are needed to evaluate longer‐term sample stability and clinical accuracy in primary screening settings.

## Author Contributions

**Kim Chu:** conceptualisation, methodology, software, validation, formal analysis, data curation, writing – original draft, writing – review and editing, visualisation, project administration. **Sofia Vidali:** investigation, data curation, writing – review and editing, project administration. **Anna Parberry:** investigation, resources, data curation, writing – review and editing. **Michelle Saull:** software, investigation, resources, data curation, writing – review and editing, project administration. **Krishna Patel:** investigation, resources, data curation, project administration. **Hannah Mohy‐Eldin:** investigation, writing – review and editing, project administration. **Laura White:** writing – review and editing, supervision, project administration. **Adam Brentnall:** conceptualisation, methodology, validation, formal analysis, writing – original draft, writing – review and editing, supervision. **Peter Sasieni:** methodology, writing – review and editing, supervision. **Rhian Gabe:** methodology, writing – review and editing, supervision. **Ranjit Manchanda:** methodology, writing – review and editing, supervision. **Jack Cuzick:** conceptualisation, methodology, writing – review and editing, funding acquisition. **Belinda Nedjai:** conceptualisation, methodology, writing – review and editing, supervision, funding acquisition.

## Funding

This study was funded by Cancer Research, UK (grant reference: PPRCPJT\100009). Laura White was funded by Barts Charity (grant reference: G‐002207).

## Ethics Statement

This study was approved by the Joint Research Management Office at Queen Mary, University of London (REC Reference 22/PR/1146). Informed consent was obtained from all participants involved in the study.

## Conflicts of Interest

K.C. reports receiving honorarium and support from SeeGene outside of the submitted work. R.M. reports research funding from YCR, Barts Charity, NHS England, GSK outside this submitted work and honorarium from GSK, MSD outside this submitted work. The other authors declare no conflicts of interest.

## Supporting information


**Figure S1:** Instructions for taking self‐samples.
**Figure S2:** Consort diagram.
**Figure S3:** Boxplots for continuous variables of (Ct score, DNA quality, DNA quantity) for each sample type and corresponding resuspension time group.
**Figure S4:** Scatterplots of DNA quality with a regression line and Pearson correlation coefficients for wet versus paired dry sample (by resuspension time group).
**Figure S5:** Scatterplots of DNA quality with a regression line and Pearson correlation coefficients for clinician versus paired dry sample (by resuspension time group).
**Figure S6:** Scatterplots of log (1 + DNA quantity) with a regression line and Pearson correlation coefficients for wet versus paired dry sample (by resuspension time group).
**Figure S7:** Scatterplots of log (1 + DNA quantity) with a regression line and Pearson correlation coefficients for clinician versus paired dry sample (by resuspension time group).
**Table S1:** Baseline characteristics for all women and by sample randomisation group and resuspension timing groups.
**Table S2:** Baseline characteristics by sample resuspension timing groups. *p*‐values are for comparisons between groups (calculated using the Wilcoxon rank‐sum test for continuous variables and Fisher's exact test for categorical variables).
**Table S3:** Number of concordant paired samples by HPV positivity.
**Table S4:** Ct values from Sample 2 by paired sample types and HPV results. Sample 2 is always HPV positive in this table.
**Table S5:** Sensitivity, specificity, positive predictive value and negative predictive value for CIN2+ detection by sample type, and resuspension time group.
**Table S6:** Summary statistics of DNA quality, Ct score, S5 score, HPV positivity, DNA quantity by urine volume.
**Table S7:** Summary statistics of DNA quality, Ct score, S5 score, HPV positivity, DNA quantity by media.

## Data Availability

The data that support the findings of this study are available from the corresponding author upon reasonable request.
