# Supplementary material for: Evaluation of the Quality and Suitability of Self‐Collected Vaginal and Urine Samples for Human Papillomavirus Testing: A Prospective Matched Study
Source: BJOG. 2026 Feb 4;133(6):1262–70. doi: 10.1111/1471-0528.70170 (PMC13040419; doi:10.1111/1471-0528.70170)
Supplement: Supplementary file 1 — Figure S1: Instructions for taking self‐samples. Figure S2: Consort diagram. Figure S3: Boxplots for continuous variables of (Ct score, DNA quality, DNA quantity) for each sample type and corresponding resuspension time group. Figure S4: Scatterplots of DNA quality with a regression line and Pearson correlation coefficients for wet versus paired dry sample (by resuspension time group). Figure S5: Scatterplots of DNA quality with a regression line and Pearson correlation coefficients for clinician versus paired dry sample (by resuspension time group). Figure S6: Scatterplots of log (1 + DNA quantity) with a regression line and Pearson correlation coefficients for wet versus paired dry sample (by resuspension time group). Figure S7: Scatterplots of log (1 + DNA quantity) with a regression line and Pearson correlation coefficients for clinician versus paired dry sample (by resuspension time group). Table S1: Baseline characteristics for all women and by sample randomisation group and resuspension timing groups. Table S2: Baseline characteristics by sample resuspension timing groups. p‐values are for comparisons between groups (calculated using the Wilcoxon rank‐sum test for continuous variables and Fisher's exact test for categorical variables). Table S3: Number of concordant paired samples by HPV positivity. Table S4: Ct values from Sample 2 by paired sample types and HPV results. Sample 2 is always HPV positive in this table. Table S5: Sensitivity, specificity, positive predictive value and negative predictive value for CIN2+ detection by sample type, and resuspension time group. Table S6: Summary statistics of DNA quality, Ct score, S5 score, HPV positivity, DNA quantity by urine volume. Table S7: Summary statistics of DNA quality, Ct score, S5 score, HPV positivity, DNA quantity by media. [file BJO-133-1262-s001.docx]

**Supplementary material**

**Figure S1:** Instructions for taking self-samples

1) Urine collection using the Colli-Pee device:

2) Copans FLOQswab (dry)


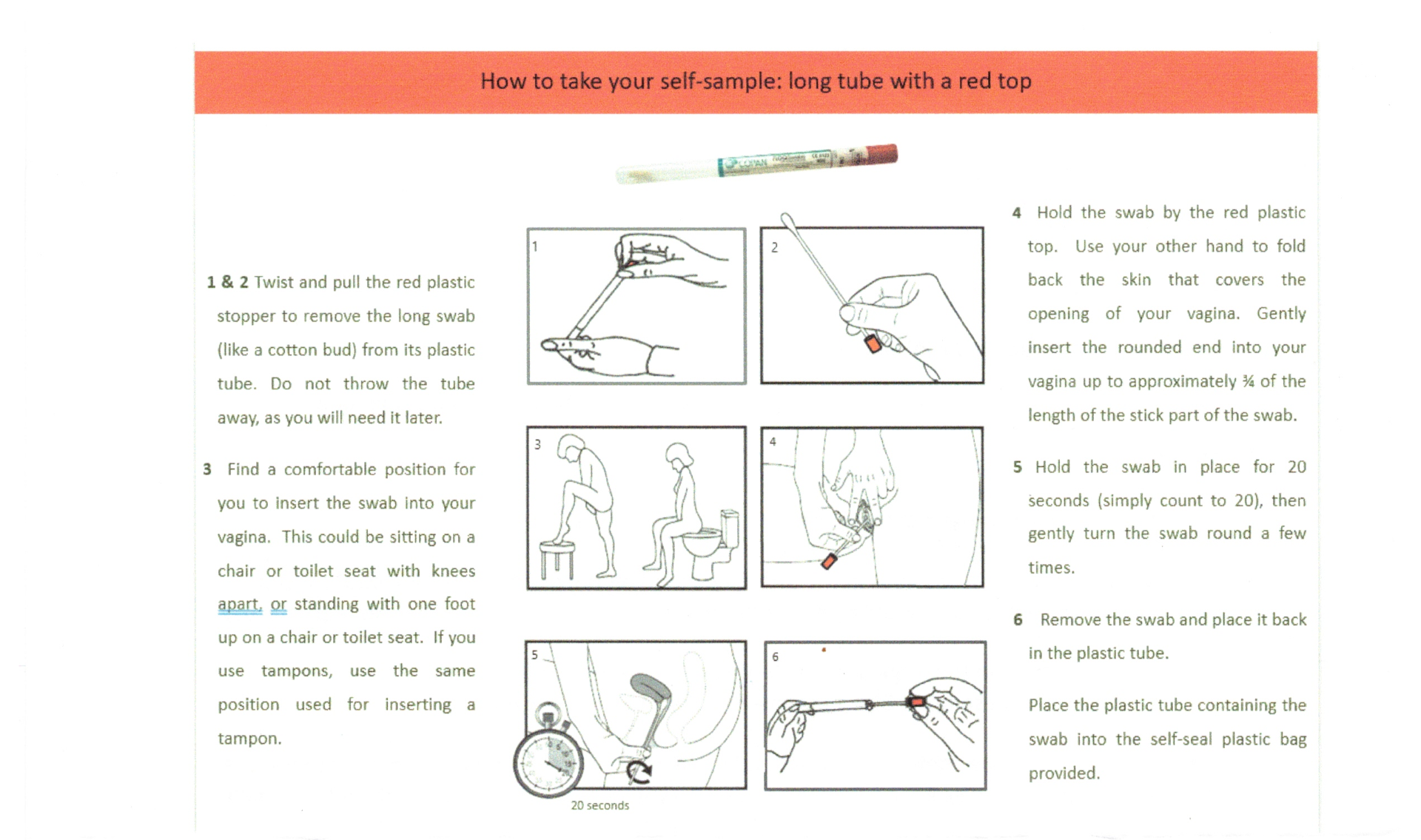


3) Copans FLOQswab (wet)


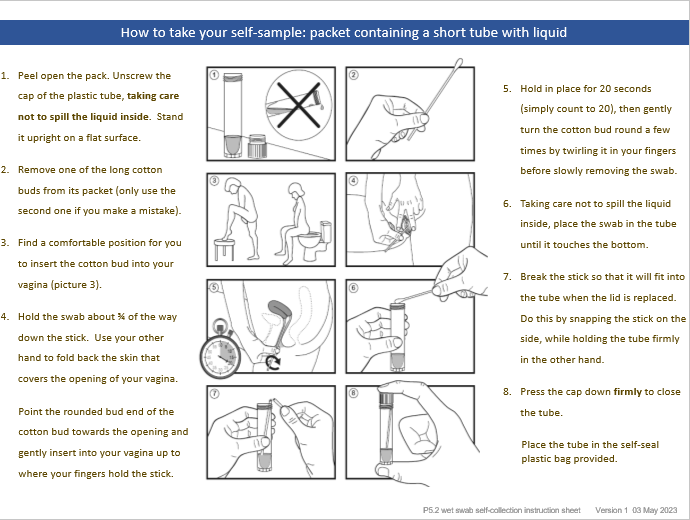


**Figure S2:** Consort diagram

**
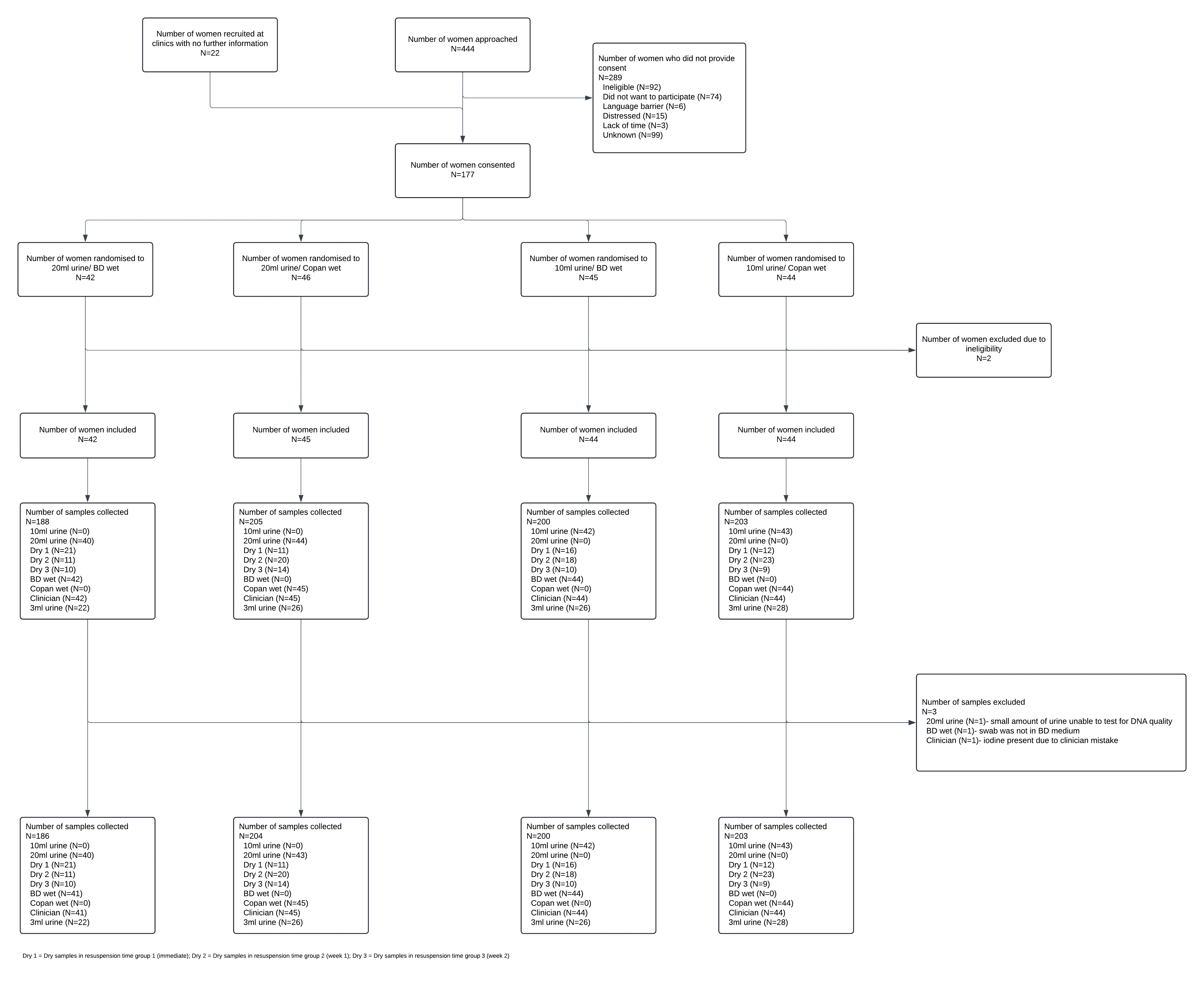
**

**Table S1: Baseline characteristics for all women and by sample randomisation group and resuspension timing groups.**

|  | **All** | **20ml urine & BD wet** | **20ml urine & Copan wet** | **10ml urine & BD wet** | **10ml urine & Copan wet** | **Time group 1 (Immediate)** | **Time group 2**  **(1 week)** | **Time group 3**  **(2 weeks)** |
| --- | --- | --- | --- | --- | --- | --- | --- | --- |
|  |  |  |  |  |  |  |  |  |
|  | N | N | N | N | N | N | N | N |
| **Total** | 175 | 42 | 45 | 44 | 44 | 60 | 72 | 43 |
|  |  |  |  |  |  |  |  |  |
|  | Median (IQR) [range] | Median (IQR) [range] | Median (IQR) [range] | Median (IQR) [range] | Median (IQR) [range] | Median (IQR) [range] | Median (IQR) [range] | Median (IQR) [range] |
| **Age at enrolment (years)** | 30 (26,35) [25,62] | 29 (27,34) [25,57] | 29 (26,35) [25,62] | 32 (26,37) [25,58] | 30 (26,36) [25,58] | 30 (26,35) [25,57] | 30 (27,36) [25,62] | 29 (27,36) [25,48] |
|  |  |  |  |  |  |  |  |  |
|  | N (%) | N (%) | N (%) | N (%) | N (%) | N (%) | N (%) | N (%) |
| **Age at enrolment (years)** |  |  |  |  |  |  |  |  |
| **<30** | 84 (48.0) | 25 (59.5) | 23 (51.1) | 18 (40.9) | 18 (40.9) | 29 (48.3) | 32 (44.4) | 23 (53.5) |
| **30-44** | 82 (46.9) | 16 (38.1) | 21 (46.7) | 24 (54.5) | 21 (47.7) | 30 (50.0) | 34 (47.2) | 18 (41.9) |
| **45+** | 9 (5.1) | 1 (2.4) | 1 (2.2) | 2 (4.5) | 5 (11.4) | 1 (1.7) | 6 (8.3) | 2 (4.7) |
|  |  |  |  |  |  |  |  |  |
| **Referral reason** |  |  |  |  |  |  |  |  |
| **S1** | 132 (75.4) | 30 (71.4) | 37 (82.2) | 32 (72.7) | 33 (75.0) | 47 (78.3) | 55 (76.4) | 30 (69.8) |
| **S2** | 26 (14.9) | 5 (11.9) | 6 (13.3) | 8 (18.2) | 7 (15.9) | 8 (13.3) | 10 (13.9) | 8 (18.6) |
| **S3** | 8 (4.6) | 4 (9.5) | 1 (2.2) | 2 (4.5) | 1 (2.3) | 2 (3.3) | 3 (4.2) | 3 (7.0) |
| **S3+** | 9 (5.1) | 3 (7.1) | 1 (2.2) | 2 (4.5) | 3 (6.8) | 3 (5.0) | 4 (5.6) | 2 (4.7) |
|  |  |  |  |  |  |  |  |  |
| **Referral cytology (prior to enrolment)**  **Unknown** |  |  |  |  |  |  |  |  |
| **Borderline** | 45 (25.7) | 13 (31.0) | 9 (20.0) | 12 (27.3) | 11 (25.0) | 14 (23.3) | 16 (22.2) | 15 (34.9) |
| **Mild dyskaryosis** | 109 (62.3) | 25 (59.5) | 33 (73.3) | 25 (56.8) | 26 (59.1) | 41 (68.3) | 43 (59.7) | 25 (58.1) |
| **Moderate dyskaryosis** | 14 (8.0) | 3 (7.1) | 1 (2.2) | 5 (11.4) | 5 (11.4) | 4 (6.7) | 7 (9.7) | 3 (7.0) |
| **Severe dyskaryosis or worse** | 6 (3.4) | 1 (2.4) | 1 (2.2) | 2 (4.5) | 2 (4.5) | 1 (1.7) | 5 (6.9) | 0 (0.0) |
| **Inadequate** | 1 (0.6) | 0 (0.0) | 1 (2.2) | 0 (0.0) | 0 (0.0) | 0 (0.0) | 1 (1.4) | 0 (0.0) |
| **Referral HPV result** |  |  |  |  |  |  |  |  |
| **Positive** | 175 (100.0) | 42 (100.0) | 45 (100.0) | 44 (100.0) | 44 (100.0) | 60 (100.0) | 72 (100.0) | 43 (100.0) |
| **Negative** | 0 (0.0) | 0 (0.0) | 0 (0.0) | 0 (0.0) | 0 (0.0) | 0 (0.0) | 0 (0.0) | 0 (0.0) |
|  |  |  |  |  |  |  |  |  |
|  | Median (IQR) [range] | Median (IQR) [range] | Median (IQR) [range] | Median (IQR) [range] | Median (IQR) [range] | Median (IQR) [range] | Median (IQR) [range] | Median (IQR) [range] |
| **Time between screening and colposcopy visit (months)** | 2.1 (1.7,2.4) [0.7,9.0] | 2.1 (1.9,2.6) [1.0,4.9] | 2.1 (1.9,2.5) [1.1,9.0] | 2.0 (1.5,2.3) [0.7,7.0] | 2.1 (1.7,2.3) [0.8,6.0] | 2.1 (1.9,2.5) [1.0,4.5] | 2.1 (1.6,2.4) [0.7,9.0] | 2.0 (1.7,2.2) [0.7,4.2] |
|  |  |  |  |  |  |  |  |  |
|  | N (%) | N (%) | N (%) | N (%) | N (%) | N (%) | N (%) | N (%) |
| **Previous treatment (more than 3 years prior to enrolment)** |  |  |  |  |  |  |  |  |
| **No** | 173 (98.9) | 41 (97.6) | 45 (100.0) | 44 (100.0) | 43 (97.7) | 59 (98.3) | 71 (98.6) | 43 (100.0) |
| **Yes** | 2 (1.1) | 1 (2.4) | 0 (0.0) | 0 (0.0) | 1 (2.3) | 1 (1.7) | 1 (1.4) | 0 (0.0) |
|  |  |  |  |  |  |  |  |  |
| **Ethnicity** |  |  |  |  |  |  |  |  |
| **Asian British** | 15 (8.6) | 5 (11.9) | 5 (11.1) | 2 (4.5) | 3 (6.8) | 5 (8.3) | 5 (6.9) | 5 (11.6) |
| **Black British** | 15 (8.6) | 2 (4.8) | 6 (13.3) | 4 (9.1) | 3 (6.8) | 4 (6.7) | 9 (12.5) | 2 (4.7) |
| **White British** | 65 (37.1) | 15 (35.7) | 18 (40.0) | 17 (38.6) | 15 (34.1) | 21 (35.0) | 28 (38.9) | 16 (37.2) |
| **Mixed** | 7 (4.0) | 2 (4.8) | 1 (2.2) | 2 (4.5) | 2 (4.5) | 5 (8.3) | 1 (1.4) | 1 (2.3) |
| **Other** | 73 (41.7) | 18 (42.9) | 15 (33.3) | 19 (43.2) | 21 (47.7) | 25 (41.7) | 29 (40.3) | 19 (44.2) |
|  |  |  |  |  |  |  |  |  |
| **IMD quintiles** |  |  |  |  |  |  |  |  |
| **1^st^ (most deprived)** | 43 (24.6) | 8 (19.0) | 10 (22.2) | 12 (27.3) | 13 (29.5) | 13 (21.7) | 22 (30.6) | 8 (18.6) |
| **2^nd^** | 82 (46.9) | 20 (47.6) | 18 (40.0) | 21 (47.7) | 23 (52.3) | 26 (43.3) | 35 (48.6) | 21 (48.8) |
| **3^rd^** | 28 (16.0) | 10 (23.8) | 11 (24.4) | 5 (11.4) | 2 (4.5) | 11 (18.3) | 8 (11.1) | 9 (20.9) |
| **4^th^** | 14 (8.0) | 3 (7.1) | 5 (11.1) | 3 (6.8) | 3 (6.8) | 5 (8.3) | 6 (8.3) | 3 (7.0) |
| **5^th^ (least deprived)** | 3 (1.7) | 0 (0.0) | 1 (2.2) | 1 (2.3) | 1 (2.3) | 1 (1.7) | 1 (1.4) | 1 (2.3) |
| **Unknown** | 5 (2.9) | 1 (2.4) | 0 (0.0) | 2 (4.5) | 2 (4.5) | 4 (6.7) | 0 (0.0) | 1 (2.3) |

S1 = Having HPV+ and cytology+ result at screening 1; S2 = Having a HPV+ and cytology- result at screen 1, and having a HPV+ and cytology+ result 1 year later at screen 2 Screen 2; S3 = Having a HPV+ and cytology- result at screen 1, 2 and 3 (all screenings are 1 year apart); S3+= Having HPV+ and cytology- result at screen 1 and 2, and having a HPV+ and cytology+ result 1 year later at screen 3; HPV = human Papillomavirus; IQR = interquartile range

**Table S2: Baseline characteristics by sample resuspension timing groups. P-values are for comparisons between groups (calculated using the Wilcoxon rank-sum test for continuous variables and Fisher’s exact test for categorical variables)**

|  | **Time group 1 (Immediate)** | **Time group 2**  **(1 week)** | **Time group 3**  **(2 weeks)** | **TG1 vs TG2** **P-value** | **TG1 vs TG3 P-value** | **TG2 vs TG3 P-value** |
| --- | --- | --- | --- | --- | --- | --- |
|  |  |  |  |  |  |  |
|  | N | N | N |  |  |  |
| **Total** | 60 | 72 | 43 |  |  |  |
|  |  |  |  |  |  |  |
|  | Median (IQR) [range] | Median (IQR) [range] | Median (IQR) [range] |  |  |  |
| **Age at enrolment (years)** | 30 (26,35) [25,57] | 30 (27,36) [25,62] | 29 (27,36) [25,48] | 0.38 | 0.76 | 0.69 |
|  |  |  |  |  |  |  |
|  | N (%) | N (%) | N (%) |  |  |  |
| **Age at enrolment (years)** |  |  |  | 0.27 | 0.56 | 0.60 |
| **<30** | 29 (48.3) | 32 (44.4) | 23 (53.5) |  |  |  |
| **30-44** | 30 (50.0) | 34 (47.2) | 18 (41.9) |  |  |  |
| **45+** | 1 (1.7) | 6 (8.3) | 2 (4.7) |  |  |  |
|  |  |  |  |  |  |  |
| **Referral reason** |  |  |  | 1.00 | 0.71 | 0.81 |
| **S1** | 47 (78.3) | 55 (76.4) | 30 (69.8) |  |  |  |
| **S2** | 8 (13.3) | 10 (13.9) | 8 (18.6) |  |  |  |
| **S3** | 2 (3.3) | 3 (4.2) | 3 (7.0) |  |  |  |
| **S3+** | 3 (5.0) | 4 (5.6) | 2 (4.7) |  |  |  |
|  |  |  |  |  |  |  |
| **Referral cytology (prior to enrolment)**  **Unknown** |  |  |  | 0.50 | 0.54 | 0.25 |
| **Borderline** | 14 (23.3) | 16 (22.2) | 15 (34.9) |  |  |  |
| **Mild dyskaryosis** | 41 (68.3) | 43 (59.7) | 25 (58.1) |  |  |  |
| **Moderate dyskaryosis** | 4 (6.7) | 7 (9.7) | 3 (7.0) |  |  |  |
| **Severe dyskaryosis or worse** | 1 (1.7) | 5 (6.9) | 0 (0.0) |  |  |  |
| **Inadequate** | 0 (0.0) | 1 (1.4) | 0 (0.0) |  |  |  |
| **Referral HPV result** |  |  |  | ND | ND | ND |
| **Positive** | 60 (100.0) | 72 (100.0) | 43 (100.0) |  |  |  |
| **Negative** | 0 (0.0) | 0 (0.0) | 0 (0.0) |  |  |  |
|  |  |  |  |  |  |  |
|  | Median (IQR) [range] | Median (IQR) [range] | Median (IQR) [range] |  |  |  |
| **Time between screening and colposcopy visit (months)** | 2.1 (1.9,2.5) [1.0,4.5] | 2.1 (1.6,2.4) [0.7,9.0] | 2.0 (1.7,2.2) [0.7,4.2] | 0.22 | 0.051 | 0.62 |
|  |  |  |  |  |  |  |
|  | N (%) | N (%) | N (%) |  |  |  |
| **Previous treatment (more than 3 years prior to enrolment)** |  |  |  | 1.00 | 1.00 | 1.00 |
| **No** | 59 (98.3) | 71 (98.6) | 43 (100.0) |  |  |  |
| **Yes** | 1 (1.7) | 1 (1.4) | 0 (0.0) |  |  |  |
|  |  |  |  |  |  |  |
| **Ethnicity** |  |  |  | 0.33 | 0.78 | 0.59 |
| **Asian British** | 5 (8.3) | 5 (6.9) | 5 (11.6) |  |  |  |
| **Black British** | 4 (6.7) | 9 (12.5) | 2 (4.7) |  |  |  |
| **White British** | 21 (35.0) | 28 (38.9) | 16 (37.2) |  |  |  |
| **Mixed** | 5 (8.3) | 1 (1.4) | 1 (2.3) |  |  |  |
| **Other** | 25 (41.7) | 29 (40.3) | 19 (44.2) |  |  |  |
|  |  |  |  |  |  |  |
| **IMD quintiles** |  |  |  | 0.19 | 0.94 | 0.36 |
| **1^st^ (most deprived)** | 13 (21.7) | 22 (30.6) | 8 (18.6) |  |  |  |
| **2^nd^** | 26 (43.3) | 35 (48.6) | 21 (48.8) |  |  |  |
| **3^rd^** | 11 (18.3) | 8 (11.1) | 9 (20.9) |  |  |  |
| **4^th^** | 5 (8.3) | 6 (8.3) | 3 (7.0) |  |  |  |
| **5^th^ (least deprived)** | 1 (1.7) | 1 (1.4) | 1 (2.3) |  |  |  |
| **Unknown** | 4 (6.7) | 0 (0.0) | 1 (2.3) |  |  |  |

S1 = Having HPV+ and cytology+ result at screening 1; S2 = Having a HPV+ and cytology- result at screen 1, and having a HPV+ and cytology+ result 1 year later at screen 2 Screen 2; S3 = Having a HPV+ and cytology- result at screen 1, 2 and 3 (all screenings are 1 year apart); S3+= Having HPV+ and cytology- result at screen 1 and 2, and having a HPV+ and cytology+ result 1 year later at screen 3; HPV = human Papillomavirus; IQR = interquartile range; ND=no difference

**Figure S3:** Boxplots for continuous variables of (Ct score, DNA quality, DNA quantity) for each sample type and corresponding resuspension time group


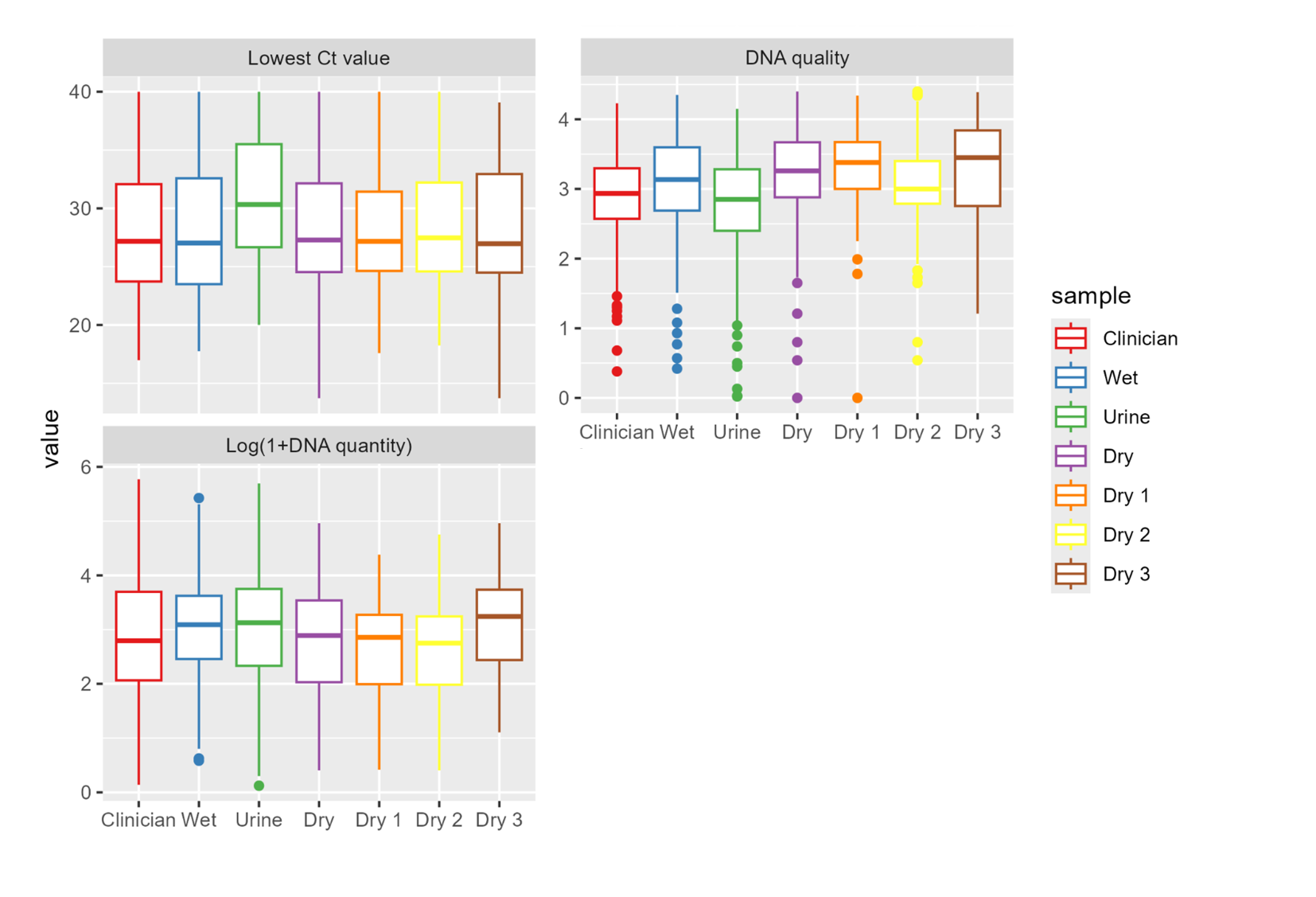


**Figure S4:** Scatterplots of DNA quality with a regression line and Pearson correlation coefficients for wet vs paired dry sample (by resuspension time group)


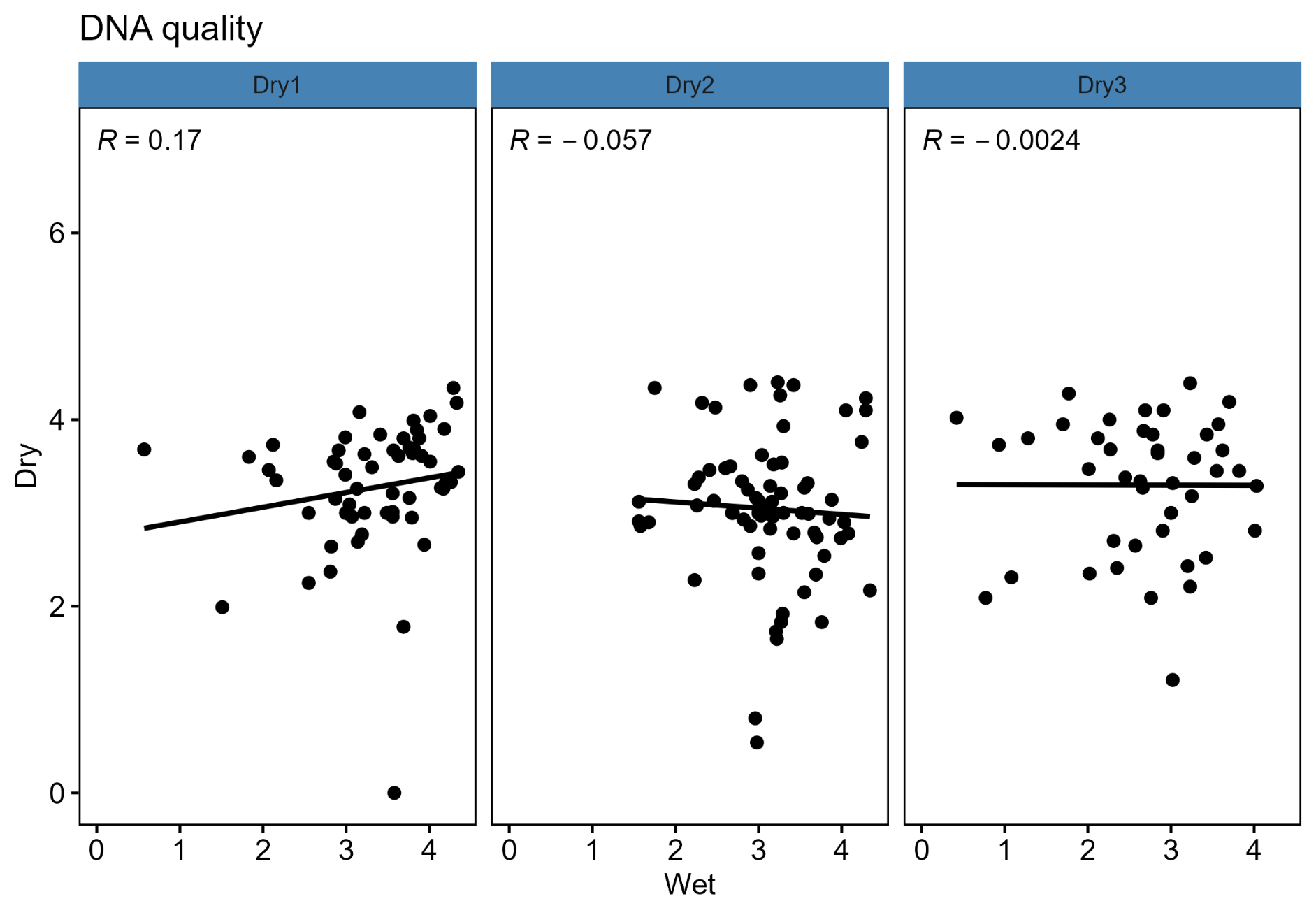


**Figure S5:** Scatterplots of DNA quality with a regression line and Pearson correlation coefficients for clinician vs paired dry sample (by resuspension time group)


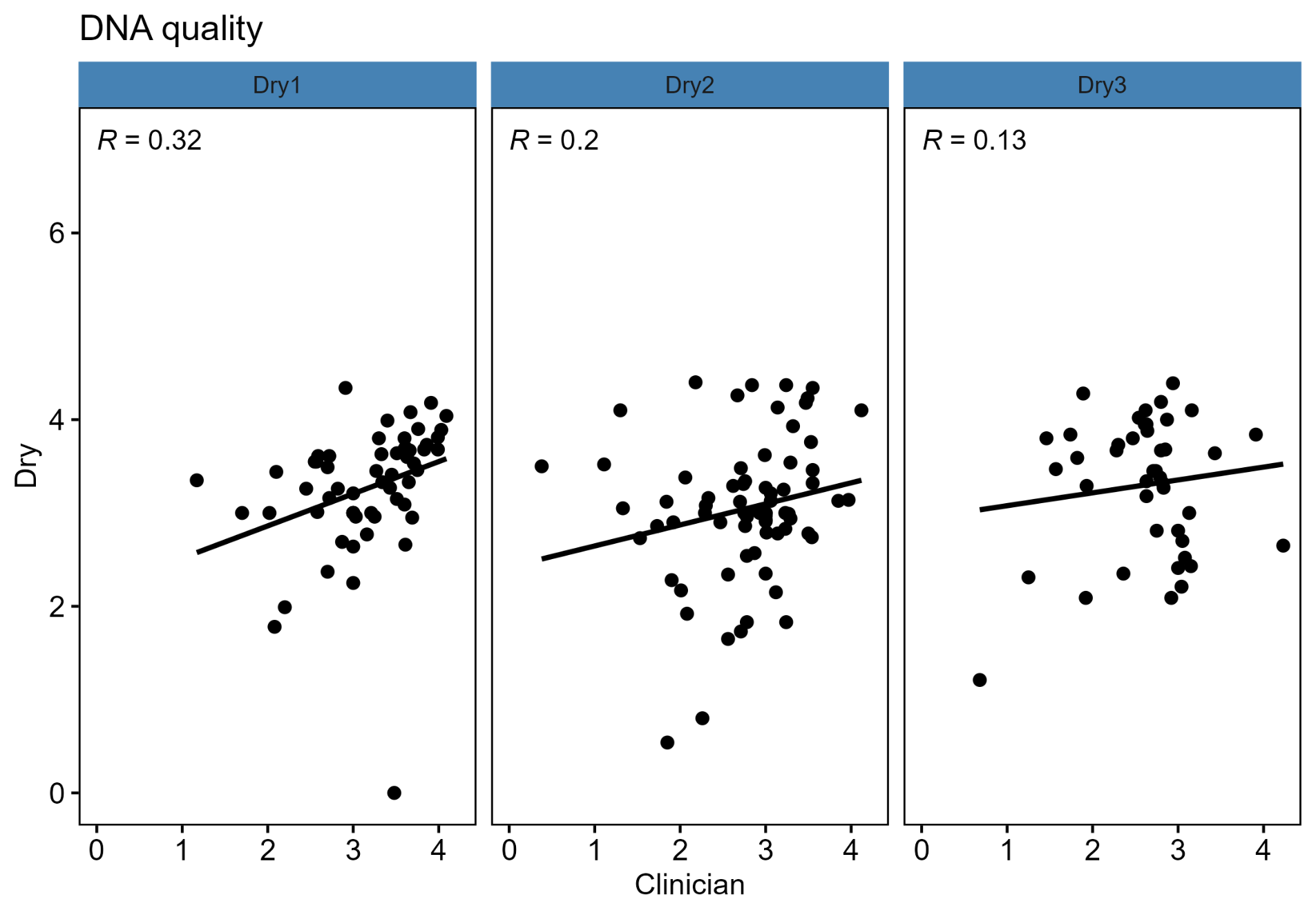


**Table S3:** Number of concordant paired samples by HPV positivity

|  | HPV +/+ | HPV +/- | HPV -/+ | HPV -/- |
| --- | --- | --- | --- | --- |
| Wet/ Dry 1 | 47 (79.7) | 1 (1.7) | 3 (5.1) | 8 (13.6) |
| Wet/ Dry2 | 55 (76.4) | 2 (2.8) | 5 (6.9) | 10 (13.9) |
| Wet/ Dry 3 | 35 (81.4) | 0 (0.0) | 1 (2.3) | 7 (16.3) |
| Clinician/ Wet | 125 (72.3) | 13 (7.5) | 15 (8.7) | 20 (11.6) |
| Wet/ Urine | 110 (66.7) | 24 (14.5) | 3 (1.8) | 28 (17.0) |

**Table S4:** Ct values from Sample 2 by paired sample types and HPV results. Sample 2 is always HPV positive in this table.

|  |  | Sample 1 |  | Sample 2 |  |
| --- | --- | --- | --- | --- | --- |
| Sample 1/ Sample 2 (HPV result) | N | Median | IQR | Median | IQR |
| Wet +/Dry 1 + | 47 | 26.0 | 23.3-28.8 | 26.1 | 24.4-28.7 |
| Wet -/ Dry 1 + | 3 |  |  | 33.3 | 33.0-33.5 |
| Dry 1 -/ Wet + | 1 |  |  | 30.8 | 30.8-30.8 |
| Wet +/Dry 2 + | 55 | 26.2 | 22.5-28.8 | 26.7 | 23.7-29.3 |
| Wet -/ Dry 2 + | 5 |  |  | 33.0 | 32.4-33.3 |
| Dry 2 -/ Wet + | 2 |  |  | 33.1 | 32.9-33.2 |
| Wet +/Dry 3 + | 35 | 25.9 | 22.6-26.9 | 25.3 | 23.8-28.6 |
| Wet -/ Dry 3 + | 1 |  |  | 33.6 | 33.6-33.6 |
| Dry 3 -/ Wet + | 0 | - | - | - | - |
| Clinician +/ Wet+ | 125 | 25.4 | 22.4-27.7 | 25.6 | 22.8-28.4 |
| Clinician -/ Wet + | 15 |  |  | 28.9 | 27.5-32.2 |
| Wet -/ Clinician + | 13 |  |  | 31.0 | 30.2-31.6 |
| Urine +/ Wet + | 110 | 28.0 | 25.3-30.4 | 25.0 | 22.2-27.2 |
| Urine -/ Wet + | 24 |  |  | 31.1 | 28.4-32.6 |
| Wet -/ Urine + | 3 |  |  | 33.5 | 33.1-33.6 |

IQR= interquartile range

**Figure S6:** Scatterplots of log(1+DNA quantity) with a regression line and Pearson correlation coefficients for wet vs paired dry sample (by resuspension time group)


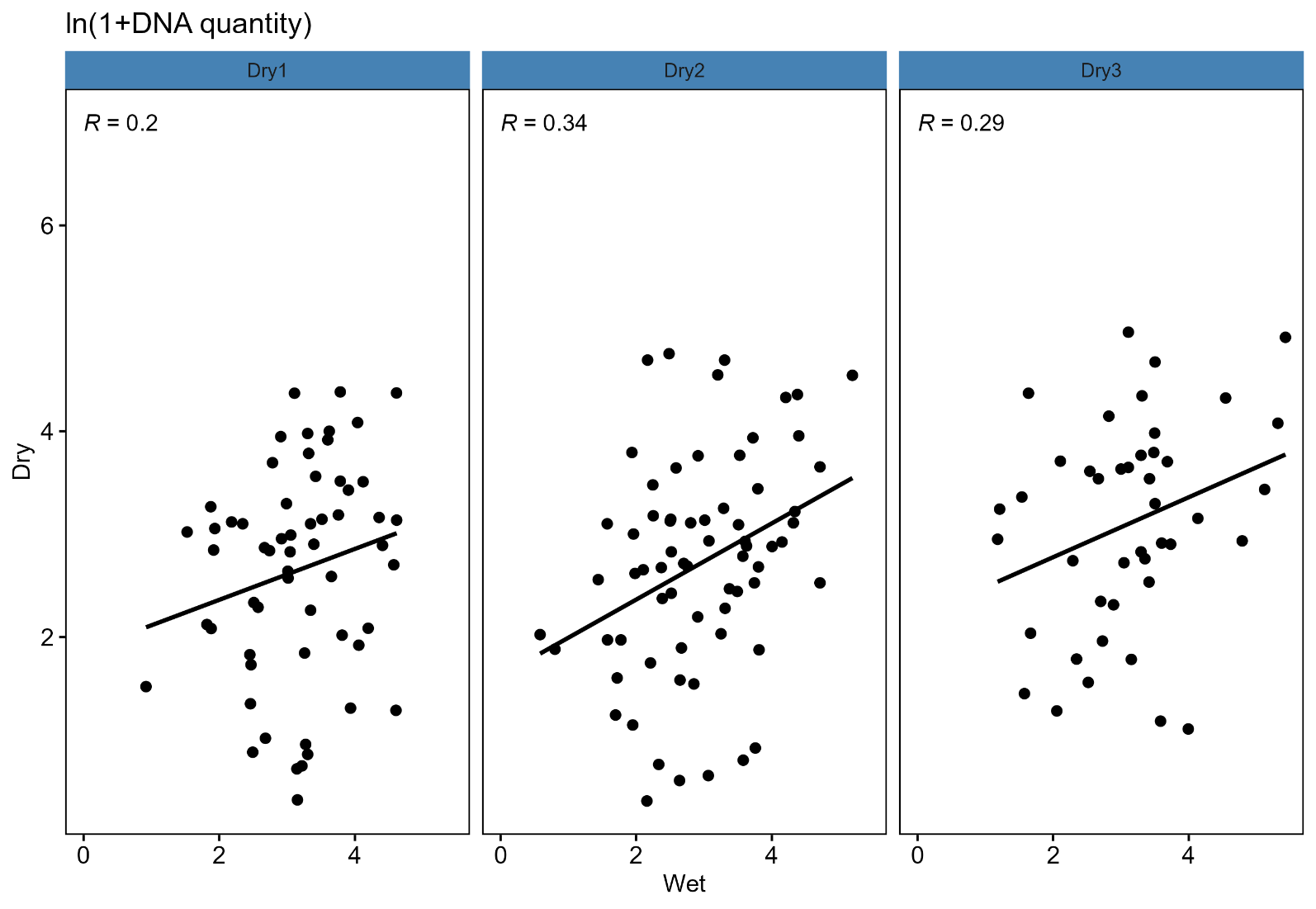


**Figure S7:** Scatterplots of log(1+DNA quantity) with a regression line and Pearson correlation coefficients for clinician vs paired dry sample (by resuspension time group)

**
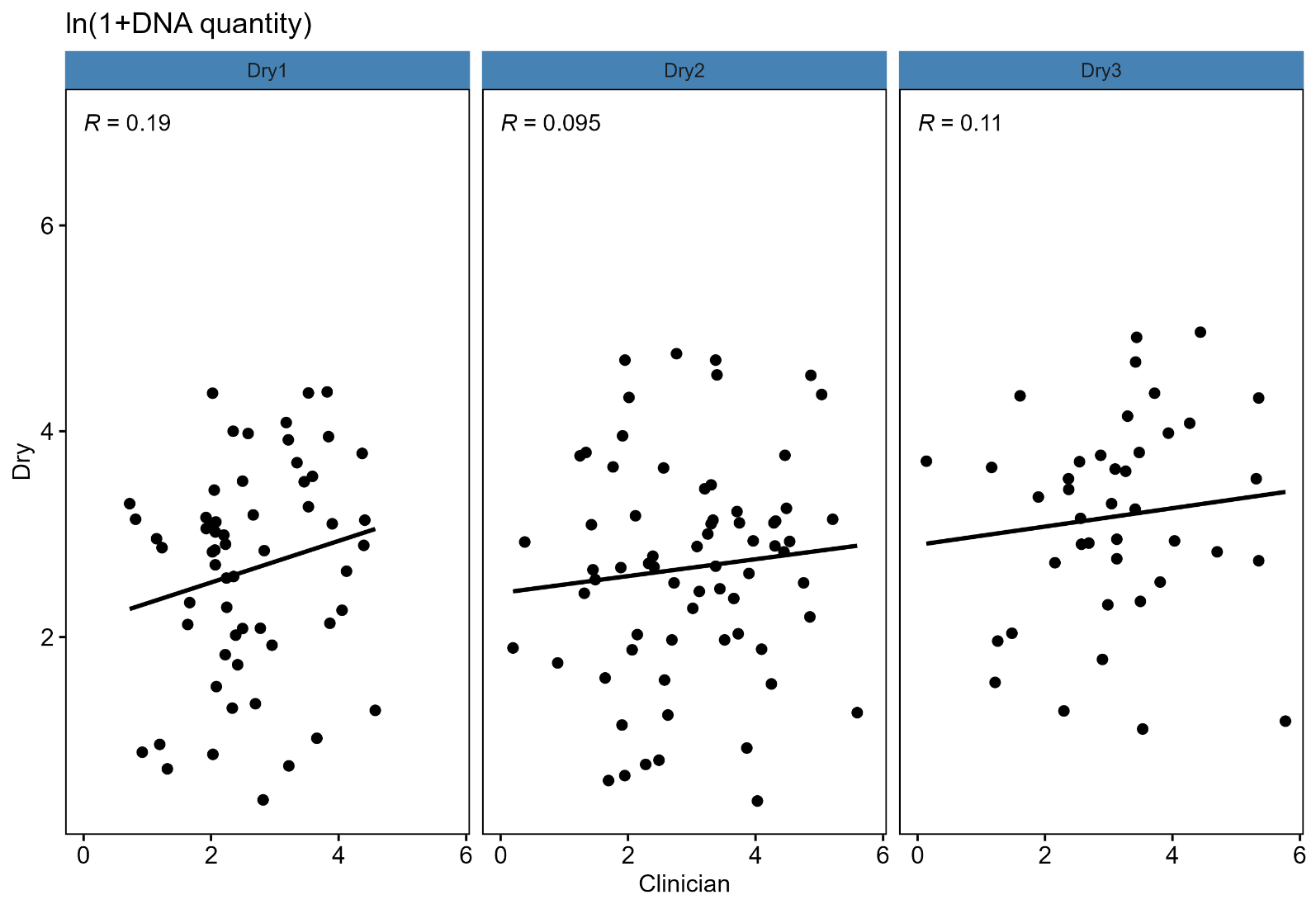
**

**Table S5: Sensitivity, specificity, positive predictive value and negative predictive value for CIN2+ detection by sample type, and resuspension time group.**

|  | Estimate [x/y] (95% CI) | | | | | | |
| --- | --- | --- | --- | --- | --- | --- | --- |
|  | Wet | Urine | Clinician | Dry | Dry time group 1 (immediate) | Dry time group 2 (week 1) | Dry time group 3 (week 2) |
| Total | 174 | 168 | 174 | 175 | 60 | 72 | 43 |
| Number of CIN2+ | 25 | 24 | 25 | 25 | 7 | 12 | 6 |
| Sensitivity | 92.0% [23/25]  (74.0-99.0) | 79.2% [19/24]  (57.8-92.9) | 92.0% [23/25]  (74.0-99.0) | 92.0% [23/25]  (74.0-99.0) | 100.0% [7/7]  (59.0-100.0) | 91.7% [11/12]  (61.5-99.8) | 83.3% [5/6]  (35.9-99.6) |
| Specificity | 21.5% [32/149]  (15.2-28.9) | 33.1% [47/142]  (25.4-41.5) | 22.1% [33/149]  (15.8-29.7) | 17.3% [26/150]  (11.6-24.4) | 17.0% [9/53]  (8.1-29.8) | 18.3% [11/60]  (9.5-30.4) | 16.2% [6/37]  (6.2-32.0) |
| Positive predictive value | 16.4% [23/140]  (10.7-23.6) | 16.7% [19/114]  (10.3-24.8) | 16.5% [23/139]  (10.8-23.8) | 15.6% [23/147]  (10.2-22.5) | 13.7% [7/51]  (5.7-26.3) | 18.3% [11/60]  (9.5-30.4) | 13.9% [5/36]  (4.7-29.5) |
| Negative predictive value | 94.1% [32/34]  (80.3-99.3) | 90.4% [47/52]  (79.0-96.8) | 94.3% [33/35]  (80.8-99.3) | 92.9% [26/28]  (76.5-99.1) | 100.0% [9/9]  (66.4-100.0) | 91.7% [11/12]  (61.5-99.8) | 85.7% [6/7]  (42.1-99.6) |

CI= confidence interval

**Table S6: Summary statistics of DNA quality, C_t_ score, S5 score, HPV positivity, DNA quantity by urine volume**

|  | **Urine 10ml** | **Urine 20ml** | **Urine 10ml vs 20ml P-value^*^** |
| --- | --- | --- | --- |
|  |  |  |  |
|  | N | N |  |
| **Total** | 85 | 83 |  |
|  |  |  |  |
|  | Median (IQR) [range] | Median (IQR) [range] |  |
| **DNA quality** | 2.84 (2.49,3.28) [0.13,3.95] | 2.89 (2.22,3.29) [0.02,4.15] | 0.53 |
| **Unknown N(%)** | 0 (0.0) | 0 (0.0) |  |
| **Lowest HPV Ct score** | 30.2 (26.3,34.9) [20.0,40.0] | 31.4 (27.1,35.9) [20.4,40.0] | 0.39 |
| **Unknown N(%)** | 0 (0.0) | 2 (2.4) |  |
| **DNA quantity** | 24.2 (11.1,42.9) [1.1,124.0] | 19.5 (7.5,39.4) [0.1,296.0] | 0.25 |
| **Unknown N(%)** | 2 (2.4) | 3 (3.6) |  |
|  |  |  |  |
|  | n/N (%) | n/N (%) |  |
| **HPV positive** | 60/85 (70.6) | 54/81 (66.7) | 0.59 |
| **Unknown** | 0 (0.0) | 2 (2.4) |  |
| **Adequate DNA quality (GQS≥1.5)** | 81/85 (95.3) | 74/83 (89.2) | 0.14 |
| **Unknown** | 0 (0.0) | 0 (0.0) |  |

GQS= genomic DNA quality score; IQR= interquartile range; ^*^P values were calculated using the Wilcoxon rank-sum test for continuous variables and differences in proportion between groups were compared using a two-sample test for equality of proportions for categorical variables.

**Table S7: Summary statistics of DNA quality, C_t_ score, S5 score, HPV positivity, DNA quantity by media**

|  | **Wet BD** | **Wet Copan** | **BD vs Copan P-value^*^** |
| --- | --- | --- | --- |
|  |  |  |  |
|  | N | N |  |
| **Total** | 85 | 89 |  |
|  |  |  |  |
|  | Median (IQR) [range] | Median (IQR) [range] |  |
| **DNA quality** | 3.07 (2.68,3.67) [0.42,4.35] | 3.14 (2.69,3.58) [0.77,4.34] | 0.75 |
| **Unknown N(%)** | 0 (0.0) | 0 (0.0) |  |
| **Lowest HPV Ct score** | 26.7 (23.5,32.0) [17.8,40.0] | 27.2 (23.5,32.7) [18.7,40.0] | 0.48 |
| **Unknown N(%)** | 0 (0.0) | 0 (0.0) |  |
| **DNA quantity** | 19.3 (8.2,33.6) [0.8,226.0] | 26.2 (11.4,41.8) [0.9,202.0] | 0.046 |
| **Unknown N(%)** | 2 (2.4) | 0 (0.0) |  |
|  |  |  |  |
|  | n/N (%) | n/N (%) |  |
| **HPV positive** | 70/85 (82.4) | 70/89 (78.7) | 0.54 |
| **Unknown** | 0 (0.0) | 0 (0.0) |  |
| **Adequate DNA quality (GQS≥1.5)** | 80/85 (94.1) | 88/89 (98.9) | 0.09 |
| **Unknown** | 0 (0.0) | 0 (0.0) |  |

GQS= genomic DNA quality score; IQR= interquartile range; ^*^P values were calculated using the Wilcoxon rank-sum test for continuous variables and differences in proportion between groups were compared using a two-sample test for equality of proportions for categorical variables.
